# Wait times for breast cancer care

**DOI:** 10.1038/sj.bjc.6603523

**Published:** 2006-12-19

**Authors:** N Saint-Jacques, T Younis, R Dewar, D Rayson

**Affiliations:** 1 Surveillance and Epidemiology Unit, Cancer Care Nova Scotia, QEII Health Sciences Centre, 560 Bethune Building, 1278 Tower Road, Halifax, Nova Scotia, Canada B3H 2Y9; 2 Division of Medical Oncology, QEII Health Sciences Centre, 471 Bethune Building, 1278 Tower Road, Halifax, Nova Scotia, Canada B3H 2Y9

**Keywords:** breast cancer, waiting times, elapsed times, care interval resolution, targeted intervention

## Abstract

Measurement of care time intervals is complex, being influenced by many factors. The definition of the care interval monitored can also bias the detection of changes in waits. The implications of using different care interval definitions to report wait times and identify delays in care provision were examined using a retrospective chart review of 637 women with surgically treated breast cancer who were referred to a cancer centre between September 1999 and 2000 or September 2003 and 2004. Overall waits between detection and adjuvant treatment increased by 12 days over the two periods, but their exact location and cause(s) could not be determined at such a low-resolution interval. At higher resolutions of care intervals, reporting the comprehensive sequence of care events, the prolongation was mainly associated with delayed access to surgery (4 days) and delivery of adjuvant chemotherapy (4 days). The latter went unnoticed when waits were reported at intermediate (referral to adjuvant treatment) and low (detection to adjuvant treatment) resolutions. Disease stage and type of first adjuvant treatment consistently and significantly influenced the length of waits. Comprehensive monitoring of the entire care path is essential to effectively prioritize interventions, assess their outcomes and optimise access to cancer care.

Prolonged wait times in the delivery of medical care are ethically, socially and politically contentious issues. They are important quality of care indicators, increasingly used to direct resource allocation and program development (Trussler, 2001; Walker and Wilson, 2001). It has become apparent that the measurement of care time intervals is complex and definition of the care interval monitored may bias the detection of change in wait times (Rayson *et al*, 2004). For example, in the United Kingdom, the introduction of a ‘guaranteed’ 2-week maximum wait for urgent referrals from general practitioners (GP) to first hospital appointment for women with breast cancer, resulted in shorter timelines for the observed interval (Robinson *et al*, 2003). However, the overall wait time from GP referral to treatment did not improve as the shorter timelines in the targeted care interval were offset by increases in others (Robinson *et al*, 2003). Wait time assessment is further complicated by the interplay of demographic, clinical and epidemiologic factors, and the need to interact with a series of health professionals working in care settings where information flow is imperfect at best (Reed *et al*, 2004; Robinson *et al*, 2005).

The inherent complexity associated with wait times requires a comprehensive examination of care time intervals within and across different care events. The aims of our study were to: (1) document wait times in breast cancer care pathways, from disease detection to start of first adjuvant therapy; (2) examine the implications of using different care intervals to report wait times and identify delays in care; and (3) assesses how clinical, demographic and treatment factors influence wait times over the entire clinical path.

## MATERIALS AND METHODS

**Figure 1:**
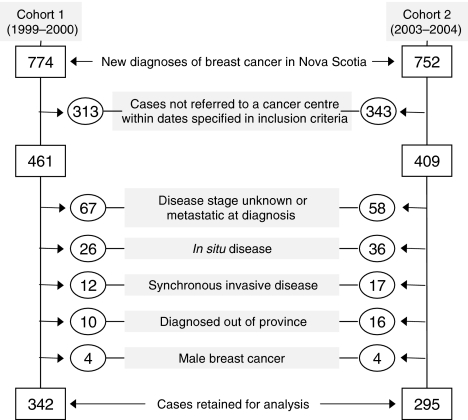
Case selection and exclusion criteria for two patient cohorts: cohort 1 (1999–2000) and cohort 2 (2003–2004). Some cases may be excluded for more than one reason.

Two cohorts of female patients were examined. Women in cohort 1 had an initial breast abnormality, detected clinically or mammographically after 1 September 1999 and were referred to one of the two regional cancer centres (Nova Scotia Cancer Centre – NSCC or Cape Breton Cancer Centre – CBCC) by 1 September 2000. Cohort 2 met the same eligibility criteria but had their initial breast abnormality detected by 1 September 2003 and were referred by 1 September 2004. All patients resided in Nova Scotia and were newly diagnosed with an invasive breast cancer for which potentially curative surgery was undertaken. Women with *in situ* (ductal or lobular) carcinoma, synchronous cancers or metastatic disease were excluded (Figure 1).

**Figure 2:**
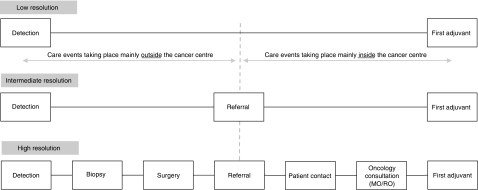
Selected levels of resolution of care intervals for breast cancer care: (1) low-resolution (long composite/overall interval embracing all events – Detection–First Adjuvant); (2) intermediate-resolution (mid-size composite intervals embracing some, but not all care events – Detection–Referral; Referral–First Adjuvant); and (3) high-resolution (small intervals embracing single/isolated care events – Detection–Biospy; Biopsy–Surgery; Surgery–Referral; Referral–Patient Contact; Patient Contact–Oncology Consultation; Oncology Consultation–First Adjuvant).

Data were obtained from radiological, surgical and pathology reports contained in the cancer centre patient chart, and a database maintained by the Nova Scotia Cancer Registry and the regional cancer centres. Dates abstracted included: (i) first clinical or mammographic evidence of breast cancer, (ii) first pathologic confirmation of invasive disease, (iii) last definitive surgery, (iv) referral receipt at one of the two regional cancer centres for consideration of adjuvant systemic or radiation therapy, (v) patient contact by cancer centre referral or physician office, (vi) first oncology (medical-MO or radiation-RO) consultation and (vii) initiation of first adjuvant (hormonal, chemotherapy, radiation) therapy. Three levels of care interval resolution were examined: low, intermediate and high (Figure 2). All wait times were expressed in calendar days.

A general linear model with a stepwise selection (*P*<0.05) was used to identify dominant cofactors influencing wait times at the three levels of care interval resolution. These cofactors included: cohort time period (Period); patient age at diagnosis (Age); area of residence (Residence); stage of disease (Disease stage); type of definitive surgery (Surgery type); type of first adjuvant therapy (First Adjuvant); distance to a cancer centre derived from address at diagnosis (Distance to cancer centre); median household income in the area of patient residence (MHI); and mean level of education in the area of patient residence (Education). Aggregate census data from 1996 and 2001 were used to compute the socio-economic factors. Days were logarithmically transformed (ln(days +1)) to better meet the assumption of data normality (Armitage and Berry, 1994). Geometric mean elapsed times and their 95% confidence intervals were estimated after correcting for all cofactors that significantly influenced wait times (i.e., the fully adjusted model including first-order interactions with Period).

The simultaneous correlations among all wait times and cofactors were summarised using Principal components analysis (Manly, 1986; Legendre and Legendre, 1998; Smith, 2002). Principal components analysis is a multivariate method that decomposes the correlations or co-variances between all variables into subsets that best capture the total variability in the data. These subsets are new composite variables (‘principal axes’) holding information from all variables of interest but largely reflecting those variables with the greatest variability and therefore the strongest influence. Dominant patterns in a data set can usually be summarised by the first two ‘principal axes’, with the primary axis (PC1) accounting for the greatest proportion of the variation in the data set; the second axis (PC2) accounting for the next largest variation, etc. The graphical presentation of these composite axes (i.e., ‘ordination’) has the feature that patients sharing similar characteristics will tend to cluster. Correlations among patients were determined from their profiles (demography, type of surgical and adjuvant treatment, etc.) and the wait they have experienced in each care interval. Patients with ‘common’ profiles or experiencing ‘average timelines’ will usually cluster near the centre of the ordination whereas patients with atypical profiles or experiencing atypical wait times (either very long or very short timelines) will be found towards the extremes of the ordination.

## RESULTS

A total of 637 women met the inclusion criteria and contributed to this study (Figure 1). Between the two cohorts, the proportion of patients residing in one region increased in the later period (Cape Breton increased from 14.3 to 23.4%; *P*=0.013; see Table 1). As well, the proportion of patients undergoing breast conservation relative to modified radical mastectomies increased over the time periods (from 40.4 to 48.3%; *P*=0.057).

**Figure 3:**
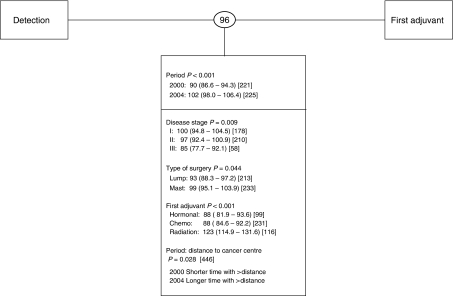
Wait times at low resolution. Shown in circles between intervals, are unadjusted geometric mean number of elapsed days. Below each interval are shown the covariates found to have a significant effect. Adjusted geometric mean number of days with 95% confidence intervals are shown in parenthesis and the sample size appears in square brackets. Period main effects are shown for every interval and interaction terms are represented by a colon. Period 2000 refers to cohort 1; Period 2004 refers to cohort 2.

When wait times were expressed at the lowest resolution (i.e., Detection–First Adjuvant), a significant prolongation in waits was observed between cohorts, increasing from 90 days to 102 days (i.e., a Period effect, *P*<0.001; Figure 3). Women living at greater distances from a cancer centre experienced an additional increase in wait times during the latter period (*P*=0.028; Figure 3). As this interval is composed of many subintervals, it was not possible to precisely identify where along the path the prolongation(s) occurred or if single or multiple events were prolonged.

Overall, for both cohorts combined, the average wait for this interval was 96 days, with 75% of the patients starting adjuvant therapy within 122 days of disease detection (Rayson *et al*, in press; Figure 3). Patients receiving radiotherapy as first adjuvant treatment experienced significantly longer care intervals relative to patients receiving chemotherapy or hormonal therapy (123 *vs* 88 or 88 days, respectively; *P*<0.001). Those diagnosed with stage I disease experienced significantly longer waits than did patients diagnosed with stage II or stage III disease (100 *vs* 97 or 85 days, respectively; *P*=0.009). Finally, those undergoing modified radical mastectomy experienced an additional 6 days before the start of first adjuvant therapy relative to patients undergoing breast conservation surgery (*P*=0.044).

**Figure 4:**
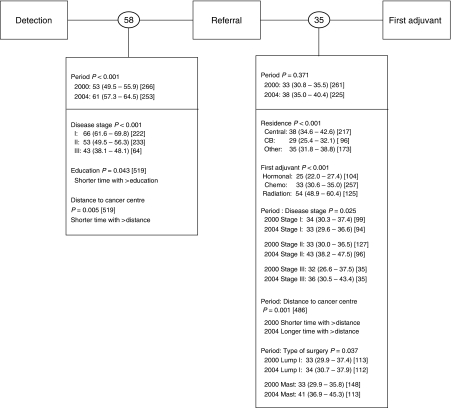
Wait times at intermediate resolution. Shown in circles between intervals are unadjusted geometric mean number of elapsed days. Below each interval are shown the covariates found to have a significant effect. Adjusted geometric mean number of days with 95% confidence intervals are shown in parenthesis and the sample size appears in square brackets. Period main effects are shown for every interval and interaction terms are represented by a colon. Period 2000 refers to cohort 1; Period 2004 refers to cohort 2.

When wait times were expressed at an intermediate resolution (i.e., Detection–Referral; Referral–First Adjuvant; Figure 4), significant prolongations were mostly associated with processes taking place outside the cancer centres (i.e., Detection–Referral). Women diagnosed in the later period waited an average of 8 additional days before referral to a cancer centre was received (*P*<0.001).

Overall, for both cohorts combined, patients waited an average of 58 days between disease detection to cancer centre referral and 35 days between cancer centre referral to the start of first adjuvant therapy (Figure 4). Patients with stage I diagnoses had significantly longer ‘Detection-Referral’ waits than those with stages II or III (66 days *vs* 53/43 days, respectively; *P*<0.001) as did patients living closer to a cancer centre (*P*=0.005). Patients from Cape Breton initiated treatment sooner than patients in Central Nova Scotia or elsewhere in the province (29 *vs* 38 or 35 days, respectively; *P*<0.001); and patients receiving hormonal therapy initiated treatment sooner than patients receiving chemotherapy or radiation therapy (25 *vs* 33 or 54 days, respectively; *P*<0.001). This interval (i.e., Referral–First Adjuvant) was marked by a change in the influence of distance to a cancer centre, disease stage and surgery type between periods: patients living further from a cancer centre (*P*=0.001) or diagnosed with stage II or III disease (*P*=0.025) or undergoing modified radical mastectomy (*P*=0.037) waited longer to initiate adjuvant therapy in the later period.

**Figure 5:**
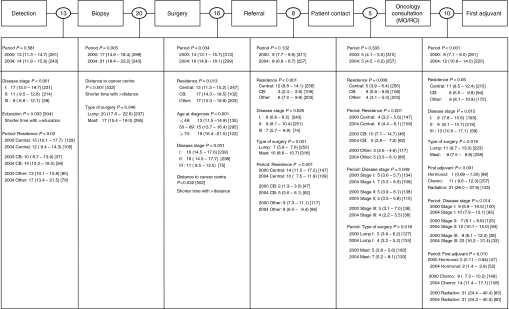
Wait times at high resolution. Shown in circles between intervals, are unadjusted geometric mean number of elapsed days. Below each interval are shown the covariates found to have a significant effect. Adjusted geometric mean number of days with 95% confidence intervals are shown in parenthesis and the sample size appears in square brackets. Period main effects are shown for every interval and interaction terms are represented by a colon. Period 2000 refers to cohort 1; Period 2004 refers to cohort 2.

When wait times were expressed at a high resolution (Figure 5), three major care events showed prolongation over time. Two of these prolongations were associated with processes taking place mostly outside the cancer centres (Biopsy–Surgery, 4 additional days; Surgery–Referral, 2 additional days) and one was associated with processes taking place mostly within the cancer centres (Oncology Consultation–First Adjuvant, 4 additional days). Factors influencing delays between the two cohorts could not be identified for the care intervals of Biopsy–Surgery and Surgery–Referral. However, the significantly prolonged wait time observed between cohorts for Oncology Consultation–First Adjuvant was associated with adjuvant hormonal or chemotherapy treatment modality (hormonal treatment: 2 *vs* 0 days; chemotherapy: 14 *vs* 9 days) and more advanced stage (stage II: 12 *vs* 7 days; stage III: 23 *vs* 9 days).

**Figure 6:**
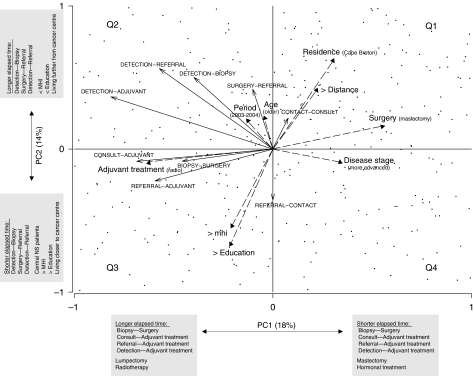
Ordination showing the inter-relationships between wait times (←) and their cofactors (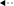). Longer arrows indicate stronger influences. Arrows pointing in the same directions indicate strong co-associations. Arrows of care intervals point in the direction of increasing wait times. Arrows for categorical cofactors point in the direction of the highest classification order as described in Table 1. PC1 and PC2 accounted for 18 and 14% of the total variance in the data, respectively. Patients (cases) appear as points. Patients clustering in the second quadrant were generally from Cape Breton, living further from a cancer centre, with lower education and income levels, undergoing lumpectomy and receiving radiation therapy as first adjuvant treatment and experiencing prolonged elapsed times in most care intervals with the exception of Referral–Patient Contact. Most wait times were oriented (increasing) in the same direction as the period effect, highlighting the general (although not necessarily statistically significant) prolongation of wait times in most care intervals.

The dominant patterns of co-associations among all wait times and cofactors are summarised in Figure 6. The first axis largely describes factors that are disease-dependent, and possibly intrinsic to the practice of a health care institution (PC1), whereas the second axis describes factors that are extrinsic to a health care institution (PC2). More specifically, PC1 suggests that patients undergoing mastectomy or receiving hormonal treatment as first adjuvant therapy generally experienced shorter waits between: Biopsy–Surgery, Oncology Consultation–First Adjuvant, Referral–First Adjuvant and Detection–First Adjuvant. Conversely, patients undergoing lumpectomy or receiving radiation as first adjuvant therapy experienced longer waits. PC2 suggests that patients served by the NSCC, living closer to a cancer centre, with higher MHI and education levels tended to experience shorter times between Detection and Biopsy, Surgery and Referral, and Detection and Referral. Conversely, patients served by the CBCC, living at greater distance from a cancer centre, with lower MHI and education levels experienced longer waits.

## DISCUSSION

Increases in waits over time were detected at all care interval resolutions. However, high resolution intervals provided the most precise determination of the location and magnitude of these increases. Intermediate resolution intervals also helped to determine whether increases in wait over time occurred within or outside the cancer centres but could not detect changes within a given interval. For example, the highly significant increase in time between oncology consultation and start of first adjuvant treatment detected at high resolution went unnoticed when intervals were resolved at an intermediate resolution (i.e., Referral–First Adjuvant). This was likely a result of an intentional compensation of one interval for delays in another (i.e., a triage effect similar to the UK case detailed in the Introduction). The lowest resolution of care intervals provided minimal information as to location or magnitude of effect, masking all useful information for targeted interventions.

Patients in the later period experienced an additional 12 days in overall care timelines largely owing to delayed access to surgery and delivery of adjuvant therapy. These points of care represent targets for discussion and potential intervention. Other key factors consistently influencing wait times were disease stage and type of first adjuvant treatment. Patients with early stage disease experienced longer waits in nearly all intervals, which may reflect the prioritization of patients with more advanced disease at diagnosis. Patients receiving radiotherapy as first adjuvant treatment also experienced longer wait times in nearly all intervals, which could partly reflect time lags associated with the planning of radiotherapy and/or prolonged referral-medical oncology consult. In our practice, the decision to proceed to radiation may be delayed until a final decision is made with regard to whether or not chemotherapy will be recommended. Therefore, wait times to a medical oncology consultation may adversely affect timelines of radiation therapy administration (see Rayson *et al*, in press). Less consistently influencing wait times were surgery type, location of patient residence and proximity of residence to a cancer centre. Education and income levels were important factors influencing wait times within a period but not between periods.

A significant increase in wait over time suggests the presence of a systemic deterioration of health care provision for women with early stage breast cancer in Nova Scotia. Significant changes over time in the influence of disease, treatment or epidemiologic variables on wait time may be early warning indicators of impending changes in health care provision. That is, they may expose an underlying structural or functional change in the care path that represents a target for proactive intervention. For example, wait time between patient contact and first oncology consultation did not vary between cohorts (period effect *P*=0.333). However, patients diagnosed with stage I disease or served by the NSCC experienced longer times to consultation in the later period, perhaps reflecting the development of a bottleneck in accessing medical oncologists.

Prospective and consistent monitoring of wait time intervals at high resolutions may be an effective method to detect delays in access to care and allow a more directed prioritisation of interventions and evaluation of their outcomes. Developing high-resolution monitoring programs might seem costly and logistically challenging, but many cancer agencies currently monitor wait times to varying degrees (Reed *et al*, 2004; Wait Time Alliance for Timely Access to Health Care, 2005; Canadian Institute for Health Information, 2006; Savage, 2006). Refining these pre-existing operational programs to a higher level of care path resolution may increase the potential for effective intervention and be more cost-effective.

This study examined the care path associated with one disease. However, system compensation between disease sites may be occurring. To obtain a more complete understanding of wait times for cancer care, an integrated, multidisciplinary approach crossing disease sites and service boundaries is required. For example, the recent indication for adjuvant chemotherapy in high-risk Non Small Cell Lung Carcinoma following curative surgery may influence a number of breast cancer care segments in some cancer centres depending on the structure of care delivery and resources. As indications for adjuvant therapy and options for palliative treatments continue to expand, we suggest that high-resolution assessment of care time intervals across all common cancers will become even more important if programmatic and resource interventions are to be targeted appropriately.

## Figures and Tables

**Table 1:** Characteristics of selected cases: Cohort 1 (1999–2000) and Cohort 2 (2003–2004)

	**Number of women (%)**	
**Characteristics**	**Cohort 1**	**Cohort 2**
*Residence*		
Central Nova Scotia	163 (47.7)	124 (42.0)
Cape Breton Island (CB)	49 (14.3)	69 (23.4)
Elsewhere in Nova Scotia (Other)	130 (38.0)	102 (34.6)
***χ***^2^ (*P*-value): 8.6 (0.013)		
		
*Age at diagnosis*		
⩽49	95 (27.8)	74 (25.1)
50–69	176 (51.5)	157 (53.2)
⩾70	71 (20.8)	64 (21.7)
***χ***^2^ (*P*-value): 0.59 (0.744)		
		
*Disease stage*		
I	152 (44.4)	124 (42.0)
II	147 (43.0)	129 (43.7)
III	43 (12.6)	42 (14.2)
***χ***^2^ (*P*-value): 0.59 (0.755)		
		
*Type of surgery*		
Lumpectomy	131 (40.4)	127 (48.3)
Mastectomy	193 (59.6)	136 (51.7)
***χ***^2^ (*P*-value): 3.6 (0.057)		
		
*First adjuvant therapy*		
Hormonal therapy	63 (21.2)	64 (26.1)
Chemotherapy	157 (52.9)	116 (47.4)
Radiotherapy	77 (25.9)	65 (26.5)
***χ***^2^ (*P*-value): 2.2 (0.331)		

χ^2^ tests of differences between cohorts and associated *P*-values are presented.
